# Population Structure and Genetic Diversity of the Pepper Weevil (Coleoptera: Curculionidae) Using the COI Barcoding Region

**DOI:** 10.1093/jisesa/ieac012

**Published:** 2022-02-27

**Authors:** D Catalina Fernández, Sherah L VanLaerhoven, Esteban Rodríguez-Leyva, Y Miles Zhang, Roselyne Labbé

**Affiliations:** 1 Department of Integrative Biology, University of Windsor, 401 Sunset Avenue, Windsor, Ontario, N9B 3P4, Canada; 2 Systematic Entomology Laboratory, USDA-ARS, c/o National Museum of Natural History, Washington, District of Columbia, USA; 3 Colegio de Postgraduados, Posgrado en Fitosanidad, Montecillo, 56100, Texcoco, Estado de Mexico, Mexico; 4 Agriculture and Agri-Food Canada, Harrow Research and Development Centre, 2585, Essex County Road 20, Harrow, Ontario, N0R 1G0, Canada

**Keywords:** pepper plant, North America, haplotype analysis, *Anthonomus eugenii*

## Abstract

The pepper weevil *Anthonomus eugenii* Cano (Coleoptera: Curculionidae) is a pest of economic importance for *Capsicum* species pepper in North America that attacks the reproductive structures of the plant. The insect is distributed across Mexico, the United States, and the Caribbean, and is occasionally found during the pepper growing season in southern Ontario, Canada. Continuous spread of the insect to new areas is partially the result of global pepper trade. Here, we describe the genetic diversity of the pepper weevil using the mitochondrial COI barcoding region across most of its geographic range. In this study, 44 (H1–H44) highly similar haplotypes were identified, the greatest number of haplotypes and haplotype diversity were observed among specimens from its native Mexico, followed by specimens from the United States. Unlike Mexico, a low haplotype diversity was found among specimens from Canada, the Dominican Republic, Italy, and the Netherlands. Out of these 44 haplotypes, 29 are reported for the first time. Haplotype diversity in the Canadian population suggests either multiple and continuous introductions of the pepper weevil into this area or a single introduction of genetically diverse individuals. We discuss the importance of such population genetic data in tailoring pepper weevil management programs, using Canada as an example.

## Resumen

El picudo del chile *Anthonomus eugenii* Cano (Coleoptera: Curculionidae) es una plaga de importancia económica para las especies de *Capsicum* en América del Norte que ataca las estructuras reproductivas de la planta. El picudo está distribuido en México, Estados Unidos y el Caribe, y se encuentra ocasionalmente durante la temporada de chiles en el sureste de Ontario, Canada. La continua propagación del picudo en nuevas áreas es en parte el resultado del comercio global de chile. En este estudio describimos la diversidad genética del picudo del chile en la mayoría de su rango geográfico usando un fragmento del gen mitocondrial COI. Se identification 44 haplotipos muy similares con el número más alto y la mayor diversidad de haplotipos en especímenes de México, seguida por los especímenes de los Estados Unidos. Una baja diversidad de haplotipos se encontró en especímenes muestreados en Canada, República Domínicana, Italia y los países bajos a diferencia de México. Veintinueve haplotipos de los 44 encontrados (H1–H44) se reportan por primera vez. La diversidad de haplotipos en la población Canadiense sugiere múltiples y continuas introducciones del picudo en ésta área o una única introducción de individuos genéticamente similars. Se discute la importancia de la genética de poblaciónes para adaptar los programas de manejo del picudo del chile usando Canadá como ejemplo.

The colonization, establishment, and dispersal of non-native insects into new environments can pose a challenge to conservation and agricultural pest management (Kenis et al. 2008, Marbuah et al. 2014, Bradshaw et al. 2016). How well non-native species establish in new environments depends in part on their potential to adapt to available hosts (Poyet et al. 2015, Fernández et al. 2021) as well as on local abiotic conditions which can otherwise restrict their survival and reproduction (Sakai et al. 2001). Despite the presence of suitable environmental conditions, ecological and biological determinants such as interspecific competition, trophic niche breadth, and life-history traits can act as selective forces creating genetic bottlenecks that are permissive only to individuals able to adapt and persist. Such selective pressure may lead to changes in the genetic makeup of the colonizing population relative to those of individuals in the native range (Lee 2002, Agosta 2006). Thus, assessing the underlying genetic variation of non-native species once they have arrived in a new environment may help identify the phenotypic traits that define a good invader and assist in devising invasive species-mitigating responses (Lawson Handley et al. 2011).

Today, a gamut of molecular tools is available to study the population genetics of non-native species. Among these, studying the sequence variation in the mitochondrial Cytochrome Oxidase subunit I (COI) barcoding gene region remains one of the most frequently used tools in species identification, delimitation (Hebert et al. 2003, Pentinsaari et al. 2014), and haplotype analyses. It is also employed for intra-population genetic differentiation studies that seek to identify how the realized geographical range of a species is affected by its behavioral or biological changes (Hu et al. 2015, Chen et al. 2016, Doorenweerd et al. 2020). Accordingly, this tool has often been used for the study of agricultural pests (Gariepy et al. 2015, 2014; Hu et al. 2015; Wang et al. 2021).

The pepper weevil, *Anthonomus eugenii* Cano (Coleoptera: Curculionidae) is a pest of economic importance to the global cultivation of *Capsicum* species. It is native to Mexico (Cano y Alcacio 1894, Van De Vossenberg et al. 2019) but is naturalized in other parts of the world including the Caribbean (Abreu and Cruz 1985, Serra et al. 2011) and the United States (Walker 1904, Campbell 1924, Elmore et al. 1934, Fullaway and Krauss 1945, Burke and Woodruff 1980, Schultz and Kuhar 2008, Ingerson-Mahar et al. 2015). The pepper weevil was briefly present but later eradicated from western Canada (Costello and Gillespie 1993), Italy (Speranza et al. 2014), and the Netherlands (Van Der Gaag and Loomans 2013), and it is sporadically found during the growing season in field and greenhouse pepper crops in southern Ontario, Canada (Fernández et al. 2017, 2020). The sporadic presence of the pepper weevil in new areas is largely explained by the extensive global trade of pepper fruit and seedlings. Developing early weevil instars transported within infested fruit or on imported pepper plant material are often intercepted at ports of entry coming from countries where the insect is native or has become naturalized (Ostojá-Starzewski et al. 2013, Van De Vossenberg et al. 2019).

In this study, we have described, analyzed, and compared the Cytochrome Oxidase Subunit I barcoding region for multiple pepper weevil individuals from six different populations across both its native and introduced ranges (i.e. Canada, Dominican Republic, Italy, Mexico, the Netherlands, and the United States). In doing so, we aimed to enhance our understanding of the movement and establishment potential for different populations of this species, as well as to assist in identifying effective management tactics based on the characteristics of the originating populations using pepper weevil in Canada as an example. This will assist global agencies in better predicting the risk of invasion of this and other invasive arthropod species in future.

## Methods

### Insect Specimen Sources

A total of 268 pepper weevil individuals (adults and larvae) were collected throughout the current geographic range of this pest in Canada (*n =* 86), Mexico (*n =* 138), and the United States (*n =* 44). Canadian specimens were obtained from infested pepper fruit collected from field and mostly greenhouse crops in 2016. Specimens from the United States were collected from infested fruit in 2018, and those from Mexico represented both preserved and freshly emerged individuals reared from field-collected fruit between 2015–2018. In addition, 127 publicly available pepper weevil mitochondrial CO1 sequences (Van De Vossenberg et al. 2019) were also included in this analysis, which added samples originally obtained from the Mexico (*n =* 22), the United States (*n =* 26), Dominican Republic (*n =* 45), Italy (*n =* 2), and the Netherlands (*n =* 32). In total, analyses were conducted in 395 sequences representing six countries and 16 populations.

### DNA Extraction and Sequencing

Whole-body DNA was extracted from pepper weevil specimens using Chelex 100 chelating resin (BioRad, Hercules, CA). Briefly, specimens were incubated in 100 µL of 5% Chelex and 2 µL of Proteinase K solution before incubation at 55°C for 24 h. Afterwards, the proteinase K was denatured by incubation of extracts at 99°C for 10 min, followed by centrifugation at 13,000 rpm for 5 min to pellet the Chelex beads. Fifty µL of the supernatant containing DNA was transferred to new tubes and DNA samples were stored at –20°C until PCR amplification. Each PCR reaction mixture included 25 µL of Froggamix 2X Plus (Froggabio, North York, ON), 1 µL of 10 µM of forward and reverse primers, 22 µL of DNA free water, and 1 µL of diluted DNA template giving a 50 µL total reaction volume. Due to changes in reagents availability, a second PCR mixture was used in later amplifications. In this case, the new PCR mixture included 0.25 µL of 10 µM of each forward and reverse primers, 2.5 µL of 10X PCR buffer, 1.25 µL of 50 mM MgCl_2_, 0.125 µL of 10 mM dNTP mix, 0.125 µL of Platinum Taq Polymerase, 1 µL of template DNA, and 19.5 µL of DNA free water giving a total reaction volume of 25 µL. Forward and reverse primers used in both mixes consisted of LCO1490: 5’–GGTCAACAAATCATAAAGATATTGG–3’ and HCO2198: 5’–TAAACTTCAGGGTGACCAAAAAATCA–3’ (Folmer et al. 1994). The amplification of the target region was verified through electrophoresis by running 5 µL of PCR product on a 1.5% agarose gel stained with RedSafe^TM^ (20,000X iNtRON) and visualized using a Gel Doc (BioRad, Hercules, CA). The remaining volume of PCR products was then purified with ExoSAP-IT (Thermo Fisher Scientific, Nepean, ON) following manufacturer guidelines and clean amplicons were bidirectionally Sanger sequenced using LCO1490 and HCO2198 primers (Robarts Research Institute, London, ON).

### Haplotype and Population Genetic Analysis

Trace files were proofread in MEGA X: Molecular Evolutionary Genetics Analysis across computing platforms (Stecher et al. 2020), and short length sequences, sequences with poor base calls, and those containing insertions-deletions were removed from any further analysis. The remaining forward and reverse directional sequences were then assembled and aligned using the progressive alignment method of ClustalW (Thompson et al. 1994) in MEGA X (Stecher et al. 2020). The Tamura 3 parameter (Tamura 1992) was found to have the best fit for nucleotide substitution using maximum likelihood. Haplotype (*h)* and nucleotide (π) diversities, and Tajima’s D statistic (Tajima 1989) were calculated for each population. Pairwise population differentiation between populations was estimated with the *G*_*ST*_ (Nei 1973). Population structures within and between each country were determined using an Analysis of Molecular Variance (AMOVA) (Excoffier et al. 1992). To more easily identify differences among populations between geographically distinct areas, samples from locations in close proximity to one another (i.e. < 600 Km) were grouped together. To this end, samples from Mexico were grouped in seven populations (northern, southern, eastern, western, central, Sinaloa, and Yucatan) and three for the United States (eastern, western, and southern). Since samples from Canada were derived from farms no greater than 300 km away from one another, they were grouped by municipality, resulting in three populations (Dresden, Kingsville, and Leamington). Samples from the Dominican Republic, Italy, and the Netherlands remained ungrouped. Analyses were performed using a combination of the following R packages: *adegenet* (Jombart and Ahmed 2011), *ape* 5.4-1 (Paradis and Schliep 2019)*, poppr* 2.9.1 (Kamvar et al. 2014), and *pegas* 0.14 (Paradis 2010). A haplotype network was assembled using the median-joining network algorithm (Bandelt et al. 1999) and plotted in Population Analysis with Reticulate Trees PopArt (Leigh and Bryant 2015). The neighbor-joining haplotype tree (Saitou and Nei 1987) with 100 bootstraps was plotted in *treeio* and *ggtree* (Yu et al. 2017, Toparslan et al. 2020, Wang et al. 2020) packages using the Hamming distance algorithm to obtain a matrix of distances between haplotypes. The rarefaction curve was plotted using haploAccum in the *spider* package (Brown et al. 2012), and the Chao1 index (Chao 1984) was calculated to estimate expected sample haplotype richness. Except where stated otherwise, these analyses were conducted in the R v 4.1.2 statistical environment (R Core Team 2021). We also performed a Bayesian clustering analysis without prior assignments in STRUCTURE v 2.3.4 (Pritchard et al. 2000). Five runs were completed for *K* = 1–6 within each clade using 10,000 burn-ins and 100,000 replicates for each run. The Evanno *ΔK* method (Evanno et al. 2005) was used in Structure Harvester v 0.6.94 (Earl and VonHoldt 2011) to determine the most likely value for *K*, and visualized the plot using Structure Plot V2.0 (Ramasamy et al. 2014).

## Results

### Haplotype Analysis

**Fig. 1.**
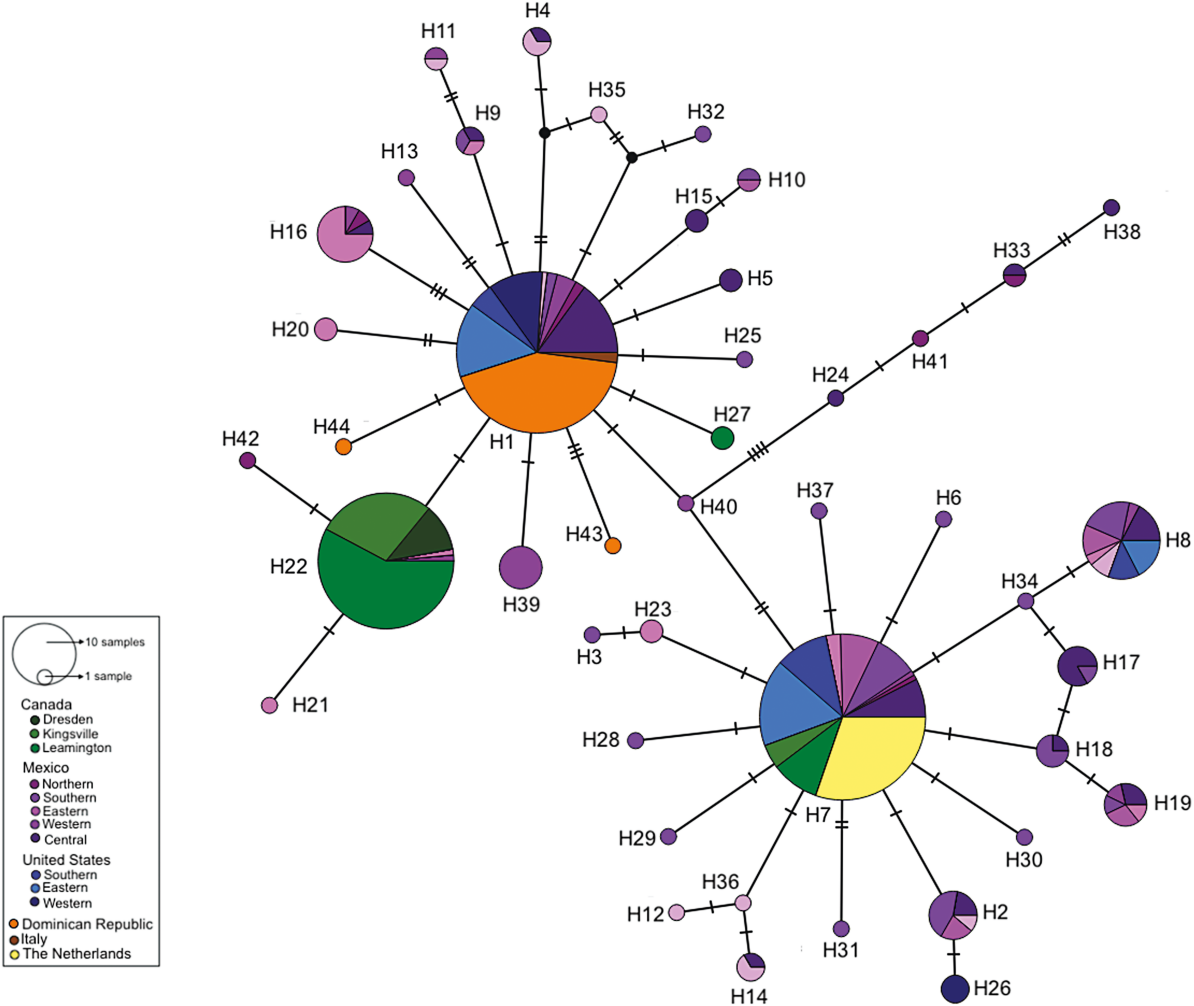
Haplotype median-joining network of the COI barcoding region (658 bp) of pepper weevil (*n =* 359) with sample locations differentiated by color. Circles (nodes) refer to the haplotypes (H1–H44) and circle size to haplotype frequency. Dashed lines in links (edges) refer to number of nucleotide differences between haplotypes. Black circles represent unsampled haplotypes.

**Fig. 2.**
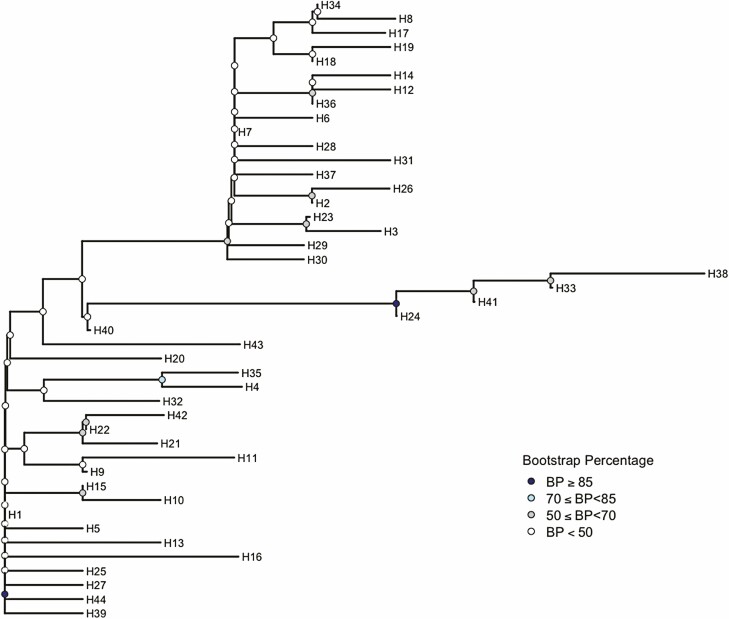
Neighbor-joining tree showing the phylogenetic relationship of 44 COI barcoding region (658 bp) haplotypes of the pepper weevil. Colored nodes represent the percentage of bootstrap confidence interval with 100 iterations.

**Fig. 3.**
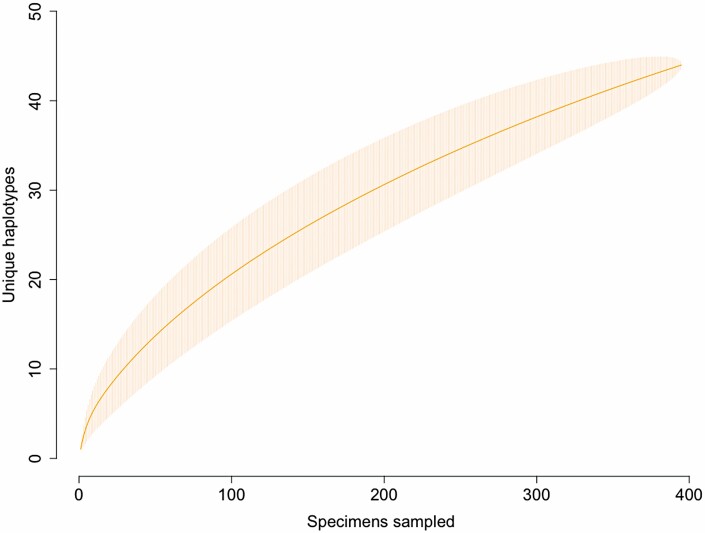
Haplotype rarefaction curve. Shaded area refers to the 95% confidence interval obtained from 10,000 permutations.

The target COI barcode region (658 bp) was successfully amplified and sequenced for 268 pepper weevil specimens (Supp. Table 1S. Accession numbers: MZ503008–MZ503275) and 127 publicly available COI sequences were also included in our analyses. Among these individuals, a total of fifty polymorphic sites were identified within the COI barcoding region, which resulted in 44 unique haplotypes (H1–H44; Table 1, Fig. 1). Of the 44 unique haplotypes, 29 are novel and previously unreported, with the most abundant being H7 (26.8%), H1 (25.3%), and H22 (17.9%). Both H1 and H7 were widely distributed among the six countries included in this study. Specimens carrying haplotype H1 originated from the Dominican Republic, the United States, Mexico, and Italy, whereas weevils with haplotype H7 were found in Canada, Mexico, the Netherlands, and the United States. In contrast, haplotype H22 was only detected from weevil populations found in Canada and Mexico. Among all haplotypes identified, a maximum variation of 12 nucleotides was detected and similarly to the network, the neighbor-joining tree with 100 bootstraps groups the haplotypes in three clusters with low branch support between them (Fig. 2). Twenty-two (50%) of the haplotypes represented in this study consisted of singletons identified only among specimens from the Dominican Republic and Mexico, with a half of all singletons detected in the southern Mexico population (i.e. states of Puebla and Oaxaca). Among North American populations, haplotype diversity (*h*) was highest in Mexican populations ranging from 0.9 to 0.7, and with 40 haplotypes out of the total 4. The Canadian populations had the lowest haplotype diversity with values of 0.3 (Table 2). Only one haplotype was present among specimens from Italy (*n* = 2) and the Netherlands (*n* = 32). The rarefaction curve, with 10,000 bootstraps did not reach an asymptote and the expected haplotype richness was of 75 haplotypes based on the Chao1 index (Fig. 3).

**Table 1.** Sample locality, sample size, and haplotypes of pepper weevil specimens amplified and used in analyses

Population	Sample size	Haplotypes
Canada		
**Dresden**		
Farm 7	8	H22
**Kingsville**		
Farm 5	5	H7
Farm 10	20	H22
**Leamington**		
Farm 1	2	H22
Farm 2	2	H7, H22
Farm 3	1	H22
Farm 4	8	H22
Farm 6	6	H22
Farm 8	11	H7, H22
Farm 9	8	H22
Farm11	15	H7, H22, H27
Mexico		
**Northern**		
Chihuahua	4	H16, H33, **H41**, **H42**
Coahuila	3	H1, H7
**Southern**		
Oaxaca	16	H2, H7, **H6**, H8, H9, H10, H19, **H25**, **H28**
Puebla	21	H1, **H3**, H7, H8, H17, H18, **H29**, **H30**, **H31**, **H32**, **H34**, **H37**
**Eastern**		
San Luis Potosi	1	H8
Tamaulipas	15	H2, H7, H8, H10, H19
**Western**		
Jalisco	16	H1, H7, **H13**, H16, H19, H22, H39
Nayarit	2	H8, H11
**Central**		
Aguascalientes	1	**H38**
Guanajuato	22	H1, H2, H7, H8, H15, H17, H18
Hidalgo	2	**H24**, H33
Queretaro	18	H1, H5, H7, H8, H9, H16
Unknown	6	H4, H8, H14, H19, **H40**
**Sinaloa**	21	H7, H8, H9, H16, H19, H20, **H21**, H22, H23
**Yucatan**	12	H1, H2, H4, H8, H11, **H12**, H14, **H35**, **H36**
United States		
**Southern**		
Florida	17	H1, H7, H8
Georgia	2	H1
**Eastern**		
New Jersey	37	H1, H7, H8
**Western**		
California	14	H1, H26
Dominican Republic	45	H1, **H43**, **H44**
Italy	2	H1
The Netherlands	32	H7

Singleton haplotypes for each population in bold.

**Table 2.** Genetic statistics of pepper weevil populations

Populations	Haplotypes	Unique Haplotypes	Haplotype diversity *h* (± SD)	Nucleotide diversity π(± SD)	Tajima’s D (*P*-value)
**Canada**					
Dresden	1	0	0	0	-
Kingsville	2	0	0.33 ± 0.09	0.002 ± 0.001	0.69 (*P* = 0.48)
Leamington	3	1	0.37 ± 0.07	0.002 ± 0.001	0.57 (*P* = 0.56)
**Mexico**					
Northern	6	2	0.95 ± 0.05	0.007 ± 0.005	–0.45 (*P* = 0.64)
Southern	19	10	0.91 ± 0.03	0.004 ± 0.002	–1.65 (*P* = 0.09)
Eastern	5	0	0.72 ± 0.09	0.003 ± 0.002	–1.28 (*P* = 0.19)
Western	10	3	0.82 ± 0.07	0.004 ± 0.002	–1.64 (*P* = 0.10)
Central	16	4	0.87 ± 0.03	0.005 ± 0.003	–1.30 (*P* = 0.19)
Sinaloa	9	3	0.8 ± 0.07	0.006 ± 0.004	–0.17 (*P* = 0.86)
Yucatan	9	3	0.95 ± 0.04	0.007 ± 0.004	–0.15 (*P* = 0.87)
**United States**					
Southern	3	0	0.60 ± 0.08	0.002 ± 0.001	0.78 (*P* = 0.43)
Eastern	3	0	0.60 ± 0.03	0.002 ± 0.001	1.48 (*P* = 0.13)
Western	2	1	0.36 ± 0.13	0.002 ± 0.002	0.53 (*P* = 0.59)
**Dominican Republic**	3	2	0.09 ± 0.06	0.0003 ± 0.0004	–1.87 (*P* = 0.06)
**Italy**	1	0	0	0	-
**Netherlands**	1	0	0	0	-
Study total	-	-	0.83 ± 0.01	0.004 ± 0.003	–1.74 (*P* = 0.08)

### Population Genetic Analysis

**Fig. 4.**
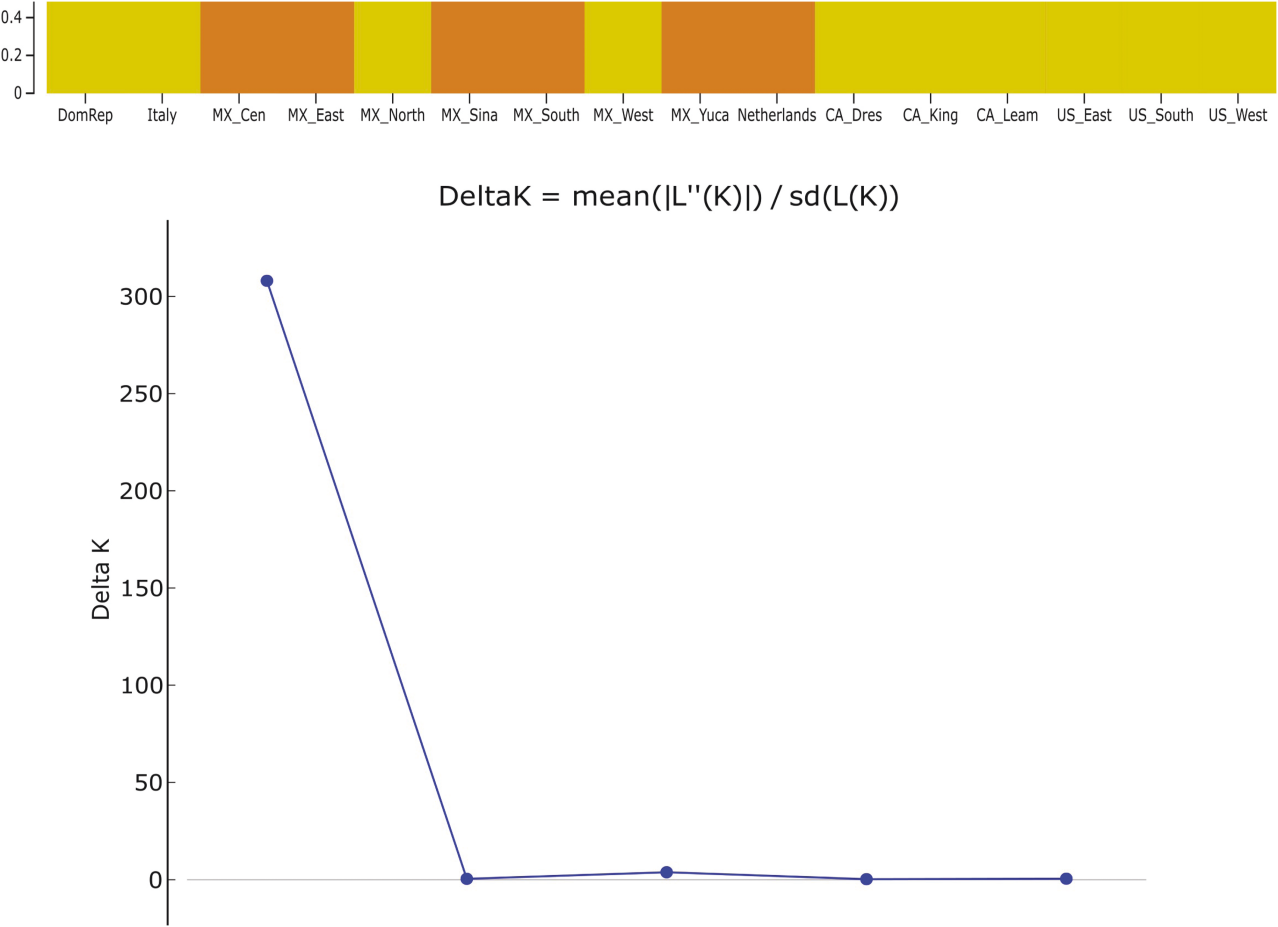
STRUCTURE plot of the most likely number of populations at *K* = 2 (top), and the *ΔK* values at *K* = 1–6 (bottom).

The overall COI nucleotide diversity identified in this study was low, although weevil populations from Mexico recorded the highest values (Table 2). The Tajima’s D estimates were not statistically different from zero for any of the populations. However, estimates were negative for all the populations in Mexico and the Dominican Republic. Positive values of the estimate were obtained for the remaining populations (Table 2). *G*_*ST*_ values of population differentiation were overall low, and maximum values were only recorded between the Canadian population in Dresden and various populations in Mexico and the United States (Supp. Table S2). The AMOVA did not detect significant genetic differentiation among the six countries (*P* = 0.06). However, significant genetic variation (*P <* 0.01) was detected among populations within countries and within populations, accounting for 11% and 77% of the total variation, respectively (Table 3). The Evanno *ΔK* method identified *K* = 2 as optimal, with a distinct grouping for most of Mexico (Central, East, Sinaloa, South, and Yucatan) along with the Netherlands, while Canada, the United States, Dominican Republic, Italy, and Northern/Western Mexico grouped together (Fig. 4).

**Table 3.** Results of an analysis of molecular variance (AMOVA) following the grouping of COI sequences from specimens with geographic proximity within countries (populations).

Source of variation	*df*	Sums of squares	Variance component	% Total variation	Ф-statistic	*P*-value
Among countries	5	153.79	0.34	12.02	0.12	0.06
Among populations within countries	11	90.01	0.31	10.77	0.12	**<0.01**
Within populations	378	824.65	2.18	77.20	0.23	**<0.01**

## Discussion

The spread of pepper weevil to new areas is facilitated by global pepper fruit trade and can result in costly outbreaks in places often unprepared to mitigate its impact. Our study adds new information relative to the population genetics of the pepper weevil. It compares the variation present in the COI mitochondrial barcoding region for this pest in both its native geographical range to those in areas where the insect has previously naturalized or is sporadically present. A previous haplotype analysis of the entire mitochondrial genome of pepper weevil specimens from Mexico, the United States, the Dominican Republic, the Netherlands, and Italy divides populations in four main haplogroups with more than 50 nucleotide differences (Van De Vossenberg et al. 2019). While this whole mitogenome haplotype analysis produced greater intraspecific diversity compared to analysis of only the barcoding region of the COI gene (Phillips et al. 2019, Fuhrmann and Kaiser 2021), our study has identified additional, previously unreported haplotypes from a broader geographical range, including Canada and additional Mexican states. In total, we now report the existence of 29 new COI haplotypes, and similar to the previously published mitogenome, this study demonstrated that Mexico retains the greatest numbers of haplotypes and overall haplotype diversity, supporting the idea that Mexico is the centre of origin for this species. In contrast, weevils belonging to populations collected elsewhere, such as from the Dominican Republic and the United States, had a smaller number of haplotypes. It is however noteworthy that haplotype diversity was considerably high among populations in the United States. This could be attributed to the possibility that multiple and independent introductions of the pepper weevil could have occurred in the United States over time, or to the longer history of presence of the pest in the country (Walker 1904, Campbell 1924, Elmore et al. 1934), compared to a more recent (2006) introduction of the weevil in the Dominican Republic (Serra et al. 2011).

Similar increases in genetic diversity have also been reported for other species with a long-term presence in invaded areas (Garnas et al. 2016). However, the contrasting haplotype composition between western and eastern pepper weevil populations in the United States suggests possibly multiple pathways of introduction to the country. Only three haplotypes were recovered among the Canadian populations, with H27 being unique to the country and H22 shared with populations from western Mexico (i.e. states of Jalisco and Nayarit) and Sinaloa. The third haplotype (H7) was the most common in the data set and it is shared with several other populations. At first, the haplotype composition in the Canadian populations suggests that pepper weevil could be the result of either multiple introductions or a single introduction event with multiple and genetically diverse individuals from its native range. However, the lack of evidence for permanent insect establishment in Canada, the ongoing trade of pepper material, and the sporadic presence of the insect during peaks of pepper growing seasons suggests that it is more likely that repeated pepper weevil introductions are occurring over time. Pepper weevil presence is most likely to remain transient in Canada as it is annually confronted with the thermal barrier present at such a northerly latitude (Fernández et al. 2020). The Netherlands and Italy populations were only represented by two of the most common haplotypes, and these two introductions were not linked (Van De Vossenberg et al. 2019). Since the Dutch and Italian pepper weevil populations have been eradicated (Van Der Gaag and Loomans 2013, Speranza et al. 2014), any future invasion is unlikely to have the same genetic composition.

The combination of a high degree of haplotype diversity but low level of divergence observed among haplotypes for pepper weevils analyzed in this study (Figs. 1 and 2), suggests that while this species is expanding its range, its populations are not rapidly diverging. Since haplotype median-joining networks place the most frequent haplotypes at the center, which are then mostly surrounded by low frequency haplotypes, a two star-like shaped network is produced. This structure is characteristic of populations undergoing expansion and supports the negative Tajima’s D estimate obtained, although the estimate was not itself significant. Additionally, the negative Tajima’s D values indicate an excess of rare haplotypes in the Mexican and the Dominican Republic populations supporting the structure of the haplotype network. The low *G*_*ST*_ values and the AMOVA also confirmed that most of the observed pepper weevil genetic diversity is found within populations, suggesting a lack of genetic differentiation across populations/panmixia. This result is consistent with the haplotype network presented in the pepper weevil mitogenome study (Van De Vossenberg et al. 2019), in which haplotypes representing different populations were mostly grouped together as part of one cluster, with the exception of haplogroup 3 which contained three highly diverse specimens from Mexico. Overall, nucleotide diversity was low and similar low values have also been reported for other insects in their native and non-native ranges (Gariepy et al. 2014). Despite supplementing our samples with these publicly available sequences, the haplotype rarefaction curve did not reach an asymptote, suggesting the specimens studied here only partially represent the total haplotype diversity and a more thorough sampling effort of pepper weevil is still required. While the Bayesian *ΔK* method identified two potential structures, it should be interpreted with care as *K* = 2 is often the recovered result due to its inability to identify panmictic populations at *K* = 1 (Janes et al. 2017, Cullingham et al. 2020).

Since the presence of the pepper weevil outside its native rage is facilitated by the global pepper trade, it is imperative to determine sources of pest origin to develop more effective management programs in new areas. Currently, pepper weevil outbreaks are largely mitigated by both the physical removal of infested fruit and plants, and through multiple applications of chemical insecticides that target exposed adult weevils on crops. However, repeated applications of insecticides are not only detrimental to well established biological control programs frequently used in greenhouse growing environments (Costello and Gillespie 1993), but are also leading to the development of resistance among field populations of pepper weevil (Servin-Villegas et al. 2008, Avendaño-Meza et al. 2015, Avendaño-Meza 2017). As an example, pepper weevils from a colony established in Ontario, Canada following a 2016 outbreak in the area showed a remarkable tolerance to multiple conventional insecticide treatments (Labbé et al. 2020). Thus, the arrival of weevils from resistant populations diminishes the overall effectiveness of management efforts within newly-invaded areas. It is, therefore, useful to understand from where invaded pests originate, particularly if their sensitivities to specific management agents differ.

Additionally, the benefits of genetically differentiating pest populations can also extend to the identification of haplotypes for insects with specific host tolerance traits. For example, we have previously shown that pepper weevil offspring development and survival decrease as levels of pepper fruit pungency increase (Fernández et al. 2021). However, specific populations of this insect species from the Yucatan peninsula of Mexico, are also known to effectively reproduce within fruit of highly pungent habanero cultivars (Berny-Mier y Teran et al. 2013, Abdala-Roberts et al. 2015). While pepper fruit pungency is variable (ranging from 100.000 to 400.000 Scoville Heat Units in habanero fruits (Canto-Flick et al. 2008), this ability to tolerate high levels of fruit pungency particular to the Yucatan population may be indicative of repeated population selection pressure. A similar case exists in the fall armyworm, *Spodoptera frugiperda* (Smith), for which different haplotypes correspond to strains that each feed and rely on distinct host plant types (Pashley 1986). Furthermore, genetic differentiation data has been collected for this (Arias et al. 2019) and other economically important insect pest species such as *Bemisia tabaci* (Gennadius) (Gauthier et al. 2014) for which knowledge of invading population sources has contributed to improving management programs for these species. For instance, the B and Q-biotypes of *B. tabaci* each correspond to distinct invasion, viral transmission, and insecticide resistance potentials (Yao et al. 2017). Taken together, this study has served to establish the groundwork for better understanding the population structure and genetic flux of an economically important pest species. We hope that similar future research will be conducted to simplify the decision-making process by regulators, following invasion of a pest into a new environment. It will also undoubtedly contribute to making more informed, effective, and sustainable pest management decisions for many invasive pest species.

## Supplementary Material

ieac012_suppl_Supplementary_Table_S1

ieac012_suppl_Supplementary_Table_S2
